# Current Status of Therapeutic Drug Monitoring in Mental Health Treatment: A Review

**DOI:** 10.3390/pharmaceutics14122674

**Published:** 2022-12-01

**Authors:** Filippo Pennazio, Claudio Brasso, Vincenzo Villari, Paola Rocca

**Affiliations:** 1 Department of Neuroscience “Rita Levi Montalcini”, University of Turin, 10126 Turin, Italy; 2 Psychiatric Emergency Service, Department of Neuroscience and Mental Health, A.O.U. “Città della Salute e della Scienza di Torino”, 10126 Turin, Italy

**Keywords:** therapeutic drug monitoring, treatment efficacy, medication adherence, schizophrenia spectrum and other psychotic disorders, bipolar and related disorders, depressive disorder

## Abstract

Therapeutic drug monitoring (TDM) receives growing interest in different psychiatric clinical settings (emergency, inpatient, and outpatient services). Despite its usefulness, TDM remains underemployed in mental health. This is partly due to the need for evidence about the relationship between drug serum concentration and efficacy and tolerability, both in the general population and even more in subpopulations with atypical pharmacokinetics. This work aims at reviewing the scientific literature published after 2017, when the most recent guidelines about the use of TDM in mental health were written. We found 164 pertinent records that we included in the review. Some promising studies highlighted the possibility of correlating early drug serum concentration and clinical efficacy and safety, especially for antipsychotics, potentially enabling clinicians to make decisions on early laboratory findings and not proceeding by trial and error. About populations with pharmacokinetic peculiarities, the latest studies confirmed very common alterations in drug blood levels in pregnant women, generally with a progressive decrease over pregnancy and a very relevant dose-adjusted concentration increase in the elderly. For adolescents also, several drugs result in having different dose-related concentration values compared to adults. These findings stress the recommendation to use TDM in these populations to ensure a safe and effective treatment. Moreover, the integration of TDM with pharmacogenetic analyses may allow clinicians to adopt precise treatments, addressing therapy on an individual pharmacometabolic basis. Mini-invasive TDM procedures that may be easily performed at home or in a point-of-care are very promising and may represent a turning point toward an extensive real-world TDM application. Although the highlighted recent evidence, research efforts have to be carried on: further studies, especially prospective and fixed-dose, are needed to replicate present findings and provide clearer knowledge on relationships between dose, serum concentration, and efficacy/safety.

## 1. Introduction

Therapeutic drug monitoring (TDM) consists of measuring drug levels in biological samples, along with a clinical and pharmacological interpretation, aiming to improve prescription appropriateness. The rationale of this clinical procedure is that a relationship between drug level, clinical effects, and toxicity can be established. TDM is usually performed on blood samples, although other biologic samples or determination of endogenous compounds related to the drug activity can be used [1]. If we just think of drugs such as digoxin, we see how TDM has marked the history of several medical treatments in past decades. In psychiatry, some drugs have a relatively long history of research and clinical application of TDM (i.e., carbamazepine, clozapine, lithium), which has become a cornerstone in guiding treatment. It is impressive how current recommendations for TDM, which are discussed below, largely overstep traditional TDM use, even if they are still too often scarcely applied in real-world clinical practice [2]. More in detail, TDM allows the determination of an individualized dose of the prescribed drug, maximizing clinical efficacy and minimizing toxicity. Appropriate clinical use of TDM requires considering both pharmacodynamic and pharmacokinetic parameters. First, under a pharmacodynamic approach, a therapeutic reference range (TRR) has to be considered for each drug, where the TRR lower limit represents the blood concentration below which a drug is unlike to have adequate clinical efficacy, while the upper limit is the concentration above which tolerability decreases or it is relatively unlikely to obtain further therapeutic improvement. Secondly, the application of another range, the dose-related reference range (DRRR), which is the expected concentration range under the prescribed dosage, permits adherence assessment and individuation of possible pharmacokinetic specificities. Thirdly, the definition of metabolite-to-parent compound ratios can be a useful tool to measure metabolizing activity.

TDM is indicated for most classes of neuropsychiatric drugs: first- and second-generation antipsychotics (i.e., haloperidol, clozapine, risperidone, olanzapine quetiapine, aripiprazole, cariprazine, etc.), mood stabilizers (i.e., lithium, valproic acid, carbamazepine, oxcarbazepine, etc.), and antidepressants (i.e., citalopram, sertraline, venlafaxine, etc.) and is recommended in a variety of clinical conditions and settings, such as suboptimal response, relapse, presence of side effects at therapeutic doses and adherence assessment. It is particularly indicated especially for drugs with a narrow therapeutic window, high inter-individual pharmacokinetic variability, or that are influenced by genetic variants of enzymes involved in drug metabolism, and in populations in which the relation between drug dose and blood level is highly unpredictable, such as limit ages, pregnancy, patients with obesity or relevant systemic diseases or treated with many different drugs [2]. In mental health, the past decades have been characterized by the introduction of several novel therapeutic interventions, comprising new drugs and an extension or more precise definition of the indications of the already existing compounds. Despite advances in the spectrum of therapeutic instruments, treatment outcomes of major psychiatric disorders remain largely perfectible. Low treatment adherence and relatively high interindividual variability of clinical efficacy and tolerability of several drugs are among the main challenges in achieving better outcomes. TDM appears to be a promising instrument to assess treatment adherence and monitor optimal drug posology. A growing body of literature supports TDM clinical implementation. Research efforts have led to an increasingly precise definition of therapeutic and dose-related reference ranges for psychotropic drugs and have highlighted the importance of combining TDM results with the evaluation of various pharmacokinetic parameters and pharmacogenetic tests [2].

Specific recommendations for TDM application in mental health have been defined, such as dose optimization, safety, adherence assessment, treatment resistance, possible drug–drug interactions (DDI), genetic alterations of drug metabolism, and physiological and clinical conditions that can lead to pharmacokinetic peculiarities. Among the latter, the main ones are limited ages (children, adolescents, and elderly), extreme body weights (i.e., obesity and severe underweight), ethnicity differences, peripartum, and pharmacokinetically relevant comorbidity (e.g., systemic infections or gastrointestinal absorption disturbances). These recommendations are summarized in the Arbeitsgemeinschaft fuer Neuropsychopharmakologie und Pharmakopsychiatrie (AGNP) Consensus Guidelines for Therapeutic Drug Monitoring in Neuro-psychopharmacology, published in 2017 [2] that represent to date the highest level of scientific evidence regarding the use of TDM in the field of mental health. TDM clinical recommendations stated in AGNP guidelines are listed in Table 1.

Specifically, AGNP guidelines propose a comprehensive evidence-based list of TRR and dose-related concentration (DRC) factors to compute DRRR for most psychoactive drugs. In addition, chronopharmacological aspects need to be considered in TDM results interpretation. TDM should be performed in the morning, before drug assumption, in order to obtain standardized and reproducible results [2]. Furthermore, a distinction between immediate- and extended-release (IR and XR) drug formulations is needed. AGNP guidelines propose specific correction parameters for DRRR calculation according to the type of formulation (IR or XR). In any case, it has to be considered that these are population-based data and, for individual patients, optimal therapeutic response and tolerability may occur at different blood levels [2]. Therefore, the definition of patients’ individual therapeutic concentration should be tailored through clinical evaluation and TDM: when a patient has reached the desired clinical outcome and no significant adverse effects are present, optimal individual drug concentration can be defined, guiding future pharmacologic treatment. In any case, TRR and DRRR enable clinicians to investigate possible causes of inadequate clinical efficacy, relapse, or adverse effects under recommended doses, comprising assessment of pseudo-resistance due to poor adherence [2].

Although the undisputed potential of TDM in psychiatry, its implementation in clinical practice is still largely heterogeneous and often insufficient [3,4,5] even for those drugs for which it has been available for many years and is highly recommended, such as lithium, valproate, carbamazepine, and clozapine [6,7,8,9,10]. Therefore, following evidence-based recommendations, more extensive employment of TDM in real-world mental health practice is desirable. A contribution in enlarging TDM use may arrive from novel mini- or not invasive sampling techniques, such as dried blood spots or oral fluid analysis, which allows simple and rapid testing, with a consequent easier applicability.

We conducted this narrative review to present the state of the art in application of TDM in mental health, with particular attention to the most recent findings issued after the AGNP guidelines. The focus on findings after 2017 aimed at providing the readers with a synthesis of what is new after the issue of AGNP guidelines, enabling a clear and simple update on the topic.

To facilitate reading, we propose the following list of contents:

**2.**  **Methods**
**3.**  **Results and Discussion**
  *3.1*  *Individual Dose Finding*
    3.1.1 Clinical Efficacy
      Schizophrenia Spectrum Disorders
      Bipolar Disorders
      Depressive Disorders
    3.1.2 Safety
      Clozapine
      Olanzapine, Other Antipsychotics, Antidepressants
  *3.2*  *Adherence*
  *3.3*  *Special Populations*
    3.3.1 Peripartum
    3.3.2 Adolescents
    3.3.3 Elderly Patients
    3.3.4 Extreme Body Weight
      Obesity
      Low Body Weight and Eating Disorders
    3.3.5 Other Medical and Surgical Conditions That Might Influence Pharmacokinetics
  *3.4*  *Drug–Drug and Drug–Smoke Interactions*
    3.4.1 Drug–Drug Interactions
    3.4.2 Drug–Smoke Interaction
    3.4.3 Other Interactions
  *3.5*  *Pharmacogenetics and TDM*
    3.5.1 Antipsychotics
    3.5.2 Antidepressants and Other Psychotropic Drugs
  *3.6*  *Novel Approaches Toward Minimally Invasive TDM*
    3.6.1 Dried Blood Spot Analysis (DBS)
    3.6.2 Volumetric Absorptive Microsampling (VAMS)
    3.6.3 Oral Fluid Analysis
    3.6.4 Other Non- or Mini-Invasive Procedures
  *3.7*  *Towards Precision Pharmacotherapy in Psychiatry*
**4.**  **Conclusions**

## 2. Materials

We searched the Web of Science database using the following search string: TS = (therapeutic NEAR/0 drug NEAR/0 monitoring) AND (TS = (mental health) OR TS = (psychiatry) OR TS = (mental disorder) OR TS = (schizophrenia) OR TS = (psychosis) OR TS = (bipolar disorder) OR TS = (depression) OR TS = (mood disorder) OR TS = (personality disorder) OR TS = (anxiety disorder) OR TS = (antipsychotics) OR TS = (mood stabilizers) OR TS = (antidepressants) OR TS = (benzodiazepines) OR TS = (haloperidol) OR TS = (chlorpromazine) OR TS = (perfenazine) OR TS = (chlothiapine) OR TS = (promethazine) OR TS = (clozapine) OR TS = (risperidone) OR TS = (olanzapine) OR TS = (quetiapine) OR TS = (paliperidone) OR TS = (aripiprazole) OR TS = (ziprasidone) OR TS = (asenapine) OR TS = (lurasidone) OR TS = (cariprazine) OR TS = (brexpiprazole) OR TS = (lithium) OR TS = (valproate) OR TS = (valproic acid) OR TS = (divalproex sodium) OR TS = (lamotrigine) OR TS = (carbamazepine) OR TS = (oxcarbazepine) OR TS = (fluvoxamine) OR TS = (fluoxetine) OR TS = (sertraline) OR TS = (paroxetine) OR TS = (citalopram) OR TS = (escitalopram) OR TS = (venlafaxine) OR TS = (duloxetine) OR TS = (bupropion) OR TS = (trazodone) OR TS = (lorazepam) OR TS = (alprazolam)). A total of 2.005 records was obtained, with a date limit of 20 October 2022. The choice to search on the Web of Science (WoS) was motivated by the fact that WoS is a platform that includes several databases, including Science Citation Index Expanded and Social Science Citation Index.

The AGNP guidelines, based on a systematic literature search, were sent for publication in May 2017 and issued in 2018 [2]. Since no significant work published before 2017 and not discussed in the AGNP guidelines emerged, we focused on research data published from 2017 on, setting data limits from 1 January 2017 to 20 October 2022.

**Figure 1:**
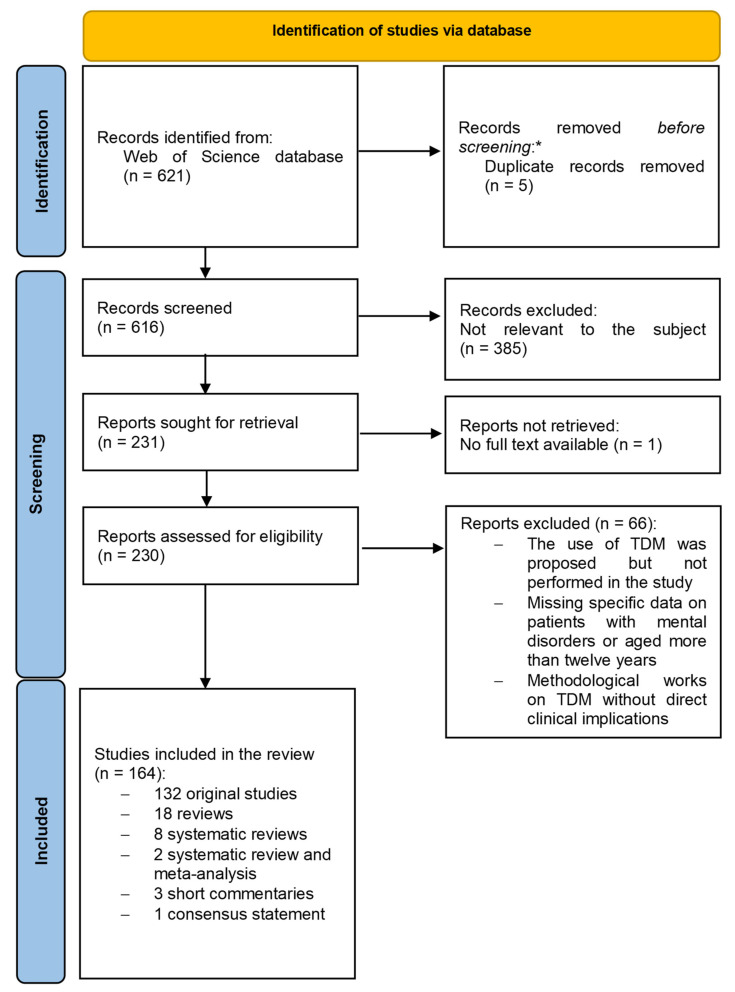
Identification and screening procedure of the studies included. * No automation tools were used.

All kinds of articles were included in the search and submitted to retrieval. We excluded articles not relevant to our review by topic (i.e., articles on technical methodologies) or clinical sample (i.e., patients with epilepsy, children aged under 12) and written in languages other than English. Records were preliminarily screened by examining the title and abstract. The full text of articles that passed initial selection underwent careful examination. The selection process is shown in Figure 1.

## 3. Results and Discussion

Following the algorithm described above and reported in Figure 1, 166 records were included in the review. Sixty-six records were excluded after full-text reading for one of the following reasons: (i) the use of TDM was proposed but not performed in the study; (ii) there were no specific or separated data on patients with mental disorders or aged more than fourteen years; (iii) the record was a methodological work on TDM without direct clinical implications. The 166 included records were: 2 systematic reviews and meta-analyses; 8 systematic reviews; 18 reviews; 2 clinical trials; 108 observational studies (59 retrospective studies, 31 prospective studies, 1 retrospective and prospective study, and 17 cross-sectional studies); 2 genome-wide association study; 6 validation studies; 11 case reports; 1 consensus statement; 1 study of device efficiency testing; 1 proof-of-concept study; 1 pilot assessment of drug urinary metabolites; and 3 short commentaries.

### 3.1. Individual Dose Finding

Since these guidelines were issued, novel research evidence has reprised the debate on TRR and DRRR for many drugs, integrating already available data with new considerations. Literature on antipsychotics, including long-acting injection (LAI) formulation and antidepressants, was largely reviewed, and novel DRRRs were proposed for clozapine, risperidone, paliperidone, and olanzapine. The studies that we have selected are mostly based on retrospective analyses of TDM databases, comprising mainly patients treated with flexible-dosing regimens, and appear not to be suitable for the determination of therapeutically effective drug concentrations because of the flexible dosing [11,12,13,14,15,16]. Therefore, fixed-dose studies are urgently needed to determine more precise relationships between dose, blood levels, and clinical efficacy. This is in line with a recent review by Hiemke, according to which a large lack of evidence on the concentration-efficacy relationship is still present [16].

#### 3.1.1. Clinical Efficacy

Clinical efficacy is the dimension representing the extent to which a patient benefits from a drug. Clinical response to treatment depends on complex interrelationships between demographic, clinical, and metabolic features and is not attributable to a single clinical or laboratory variable. TDM represents a valuable and still underemployed tool to understand the mechanisms involved in the interplay between such factors, both at population and individual levels, also with the potential to integrate all variables in vivo for every patient. In addition, TDM can be helpful in identifying low drug serum concentration, possibly due to individual pharmacokinetic variants (demographic, genetic, linked to concomitant diseases or treatments) as a factor involved in resistance. Since the pharmacological treatment of mental disorders is mainly carried out with oral therapies taken by the patient at home, and there are no methods to predict with certainty what will be the minimum effective dose for that patient, TDM can help to find such a dose. Consequently, it may promote the obtainment of therapeutic effects by minimizing the adverse ones, thereby reducing the risk and duration of hospitalization as well as mental health costs. This is in agreement with AGNP guidelines that indicate the TDM in relapse and recurrence prevention and management, lack of clinical improvement under recommended doses, and the determination of optimal individual drug concentration when the patient has attained the desired clinical outcome in many mental disorders [2].

##### Schizophrenia Spectrum Disorders

FEP is a very challenging issue for psychiatrists and a crucial period in the management of the disease: an efficient early treatment enables better long-term treatment outcomes. Although research data are still scarce and controversial, TDM appears to be a promising tool in optimizing treatment from its beginning. Additionally, for the current review, we found incoherent evidence. In particular, an observational study on 64 patients with FEP receiving second-generation antipsychotics reported no significant correlation between serum concentrations and clinical effects, while data drawn from a large multicentric clinical trial and regarding 47 patients receiving olanzapine evidenced a role for TDM in predicting clinical efficacy after two months [17,18].

Early clinical response evaluation and dose-finding are critical issues also in schizophrenia relapse and maintenance. Data from a prospective study on aripiprazole suggest the utility of measuring the plasma level of aripiprazole and its active metabolite dehydroaripiprazole after a week of treatment, while another performed on Asian patients from Taiwan reported a higher clinical response with aripiprazole serum concentrations higher than the TRR proposed for the western population [19,20]. A recent meta-analysis suggests a high inter-individual variability and the influence of CYP2D6 genotypes in the treatment of schizophrenia spectrum disorders with aripiprazole and proposes a low daily dose approach with a starting dose of 10 mg and 5 mg for known poor metabolizers [21]. A prospective study on patients receiving second-generation antipsychotics included in a naturalistic drug-monitoring program retrieved no relationship between early clinical response and drug plasma level [22]. Another naturalistic and flexible-dose study conducted on patients treated with risperidone enlightened a possible association between risperidone active moiety (risperidone and its active metabolite 9-hydroxyrisperidone, that is paliperidone) plasma concentrations and clinical response [23]. As for FEP, available evidence on TDM in schizophrenia relapse is still sparse and heterogeneous [19,20,21,22,23], possibly due to the design of the studies that considered flexible, individualized dosage. However, even if real-world data are relatively lacking and highly interesting on this topic, studies with fixed-dose regimens are still awaited to define more reliable correlations between dose, blood concentration, and efficacy.

Treatment resistance in schizophrenia is a therapeutic challenge, and identifying factors associated with inadequate clinical response is often complex. TDM and early pharmacogenetic testing may help to disentangle factors underlying clozapine resistance through the analysis of genetic variants of metabolizing enzymes, blood–brain barrier transporters, and receptors of neurotransmitters [24,25,26,27,28,29,30]. Twelve studies focused on treatment resistance or failure in schizophrenia [17,24,30,31,32,33,34]. In particular, a retrospective analysis of a large sample of patients included in the Clinical Antipsychotic Trials of Intervention Effectiveness (CATIE) study and randomized to olanzapine or risperidone revealed a significant correlation between low antipsychotic blood levels and treatment failure [31]. Other retrospective data on patients evaluated as treatment-resistant by their clinician and switched to clozapine—the gold standard therapy for treatment-resistant schizophrenia—or to long-acting injectable (LAI) antipsychotics revealed a relatively high prevalence of undetectable or subtherapeutic blood levels of oral antipsychotics [32,33]. A solid correlation between clozapine serum concentration and dose-adjusted serum concentration clinical response has been confirmed by several studies [17,24,25]. On the other hand, a review assessing the clinical utility of clozapine/norclozapine ratio found no significant association with clinical response, while a subsequent clinical study on 50 patients observed an association between a higher clozapine/norclozapine ratio and clinical non-response [26,27]. Two studies demonstrated that TDM can also be useful in maintaining efficacy and safety when switching from a clozapine formulation to another, permitting a timely assessment of possible scarcely predictable intra-individual differences in blood levels due to the switch to a novel formulation [29,30]. Finally, another study showed that TDM may also assist clinicians in exploring unusual therapeutic strategies in treatment-resistant patients, such as ensuring safety in the case of prescription of off-label dosage of quetiapine [34].

Finally, since point-of-care personnel normally administer LAI antipsychotics, the role of TDM for this kind of treatment is usually underestimated and routinely less prescribed than for oral treatment, as adherence assessment is not needed. However, despite the certainty of drug assumption, the problem of not reaching adequate blood concentration due to pharmacokinetic processes concerns patients treated with LAI as well as those receiving oral treatment [12,35,36,37,38,39]. Furthermore, TDM can be particularly helpful during the first phase of treatment with LAI antipsychotics since, for these formulations, the achievement of the steady state may take some months [35]. In particular, one study suggested that TDM can be useful when switching from oral to LAI formulation [11]. Another study demonstrated that under-range atypical antipsychotics serum concentrations after LAI administration predicted psychotic relapse [39]. Focusing on the first phase of treatment with paliperidone LAI formulations, an observational study reported that monthly paliperidone palmitate injection (PP1M) may take 8 months to reach the steady state and 3-month paliperidone palmitate injection (PP3M) take over 1 year [35]. Finally, four studies suggested to performed TDM with LAI formulations of antipsychotics when individual dose-finding might be difficult because of high inter-individual variability between patients [12,35,36,37]. This is the case of extreme age, extreme BMI, and altered cytochrome CYP2D6 activity [12,35,36,37].

Novel antipsychotic cariprazine pharmacokinetics needs a brief focus. Cariprazine has two active metabolites: desmethylcariprazine (DCRP) and didesmethylcariprazine (DDCRP), both pharmacologically equipotent to the maternal drug. Cariprazine and DCRP half-lives are of about 1–2 days, while DDCRP has a 2–3-week half-life, resulting in several times higher systemic exposure; cariprazine and DCRP steady states are reached on average after 2 weeks of treatment, while DDCRP steady state could need up to 8 weeks or more to be obtained, with high inter-individual variability [40]. AGNP guidelines propose TRR and dose-related concentration factors to compute DRRRs for cariprazine and its active metabolites, considering pharmacokinetic parameters, which have been subsequently reported by a specific literature review [40]. Moreover, a recent large clinical study on 2558 patients, which assessed the correlation of plasma levels of cariprazine and its active metabolites with clinical outcomes and adverse effects, confirmed the validity of the dose range recommended for schizophrenia (1.5–6 mg/d) and for bipolar mania (3–6 mg/d), in agreement with previous therapeutic drug monitoring data [41].

##### Bipolar Disorders

Regarding mood-stabilizing drugs, namely lithium, valproic acid, carbamazepine, and lamotrigine, TRRs are based on consolidated evidence, and TDM is highly recommended and employed for clinical decision-making [2]. Lithium stands as a singular fortunate case for which relationships between serum concentration and clinical effects are well established. A clear association between clinical response in any specific phase of bipolar disorder and blood level is defined and is of great clinical utility. Higher serum concentrations are indicated in the management of manic episodes and lower levels in depressive phases, while optimal maintenance values have to be individually determined and generally represented by low/intermediate concentration within the frame of the TRR [42]. Many atypical antipsychotics are effective in treating acute depressive or (hypo)manic episodes and as maintenance treatment and are therefore extensively prescribed for bipolar disorders. We found some evidence that TDM may help in characterizing optimal drug plasma levels according to the disease phase, as already defined for lithium [43]. In particular, about this topic, we found exclusively a prospective study conducted by Mauri and colleagues [43] on aripiprazole LAI and paliperidone LAI in patients affected by bipolar disorder type I with manic predominance. This study demonstrated that lower paliperidone plasma levels and intermediate aripiprazole plasma levels had a positive effect on depressive symptoms, which may be present and clinically relevant even in patients with this specific subtype of bipolar disorder.

##### Depressive Disorders

Evidence on TDM of antidepressant drugs is largely explored in the AGNP consensus guidelines [2] and in a recent meta-analysis [44], and in two reviews that extensively analyzed significant literature on most antidepressants [44,45]. Specific therapeutic reference ranges are proposed for each drug [2,45], even if a clear linear correlation between blood levels and clinical response is unclear for most available drugs [2,44,45]. Nevertheless, a prospective observational study recently demonstrated a significant bell-shaped quadratic relationship between the efficacy of first-line antidepressants serum concentrations of those drugs, suggesting a progressive increase in antidepressants efficacy up to around the upper normalized limit of the TRR with a decrease in the antidepressant response at higher serum concentrations [46]. Furthermore, two recent works on venlafaxine conducted in a naturalistic setting showed an association between clinical response and the sum of serum concentration of venlafaxine and its active metabolite O-desmethylvenlafaxine, with a progressive increase in antidepressant efficacy up to the concentration of 400 ng/mL and a decrease at higher blood levels, suggesting that TDM, more than oral dosage, can represent the major tool for optimizing venlafaxine treatment [47,48]. Despite this recent evidence, further research with fixed doses [44] and real-world data are needed to provide clinicians with more precise and clinically relevant instruments for employing the TDM of antidepressants.

Original articles on TDM application in optimizing clinical efficacy are summarized in Table 2.

#### 3.1.2. Safety

As stated in the AGNP consensus guidelines, TDM is mandatory for safety reasons for drugs with a narrow therapeutic window and/or with high risk related to overdosage, such as, for example, lithium, carbamazepine, or clozapine [2]. AGNP guidelines also provide, aside from TRR values, useful laboratory alert levels for each drug, such as, for example, 1000 ng/mL for clozapine, 1.2 mmol/L for lithium, or 20 μg/mL for carbamazepine. For other drugs, in routine practice, it is often the clinician’s judgment to guide dose reduction or discontinuation because of adverse effects. However, blood levels can address proper dose-finding or appropriate discontinuation more precisely and earlier, improving treatment safety [2,11,13]. In our search, we found twelve original contributions that we present in this order: clozapine, olanzapine, other antipsychotics, and antidepressants.

##### Clozapine

A large real-world observational study, including 874 patients conducted after the introduction of a specific monitoring policy, showed TDM utility in individuating patients with high-risk clozapine blood concentrations [50]. Furthermore, as reported in a case report [51], easy point-of-care testing may improve clozapine TDM implementation and prevent lethal events. One study focused on the risk of neutropenia, revealing a negative relationship between norclozapine levels and neutrophil granulocyte count [52]. Regarding neurologic side effects, a multicenter cross-sectional study confirmed the validity of the TRR proposed by the AGNP guidelines and found greater efficacy when reaching clozapine serum concentrations over 1000 ng/mL, accompanied on the other hand by significantly higher central nervous system toxicity (seizures, myoclonus, sedation) [24]. Moreover, the correlation between serum concentration and seizures has also been confirmed by a recent literature review [19]. Clozapine blood levels and BMI appeared to be linked by a bi-directional relationship: on the one hand, high clozapine blood levels are associated with weight gain and insulin resistance, especially in overweight patients; on the other hand, elevated BMI, which can arise consequently to treatment, is associated with an increase in plasma concentration, probably because clozapine may deposit in body fat, leading to a consequent reduction in clearance [53]. Finally, clozapine blood concentration appeared to be positively associated with sialorrhea [54]. In conclusion, although a debate on reference range is still ongoing, TDM may also be useful in the monitoring of adverse effects [55], such as neutropenia [52], seizures [24], and sialorrhea [54].

##### Olanzapine, Other Antipsychotics, Antidepressants

Higher levels of olanzapine serum concentration correlated with weight gain after a two-month treatment in patients with FEP, and metabolic dysfunction was more severe and dose-dependent in drug-naive patients as compared to patients with chronic schizophrenia [18,56]. Two studies focused on the association between N-desmethyl-olanzapine (DMO), an olanzapine metabolite, and metabolic side effects [53,57], suggesting a protective role of this molecule in the development of these dangerous adverse effects. In a recent study about different drugs, higher serum antipsychotics concentrations were significantly associated with lower subjective well-being, while with paliperidone palmitate one month (PP1M), a higher serum concentration/LAI dose ratio was associated with a higher risk of developing adverse effects [58]. Focusing on specific results of this review, recent evidence demonstrated that TDM could prevent or reduce metabolic adverse effects of antipsychotics by helping to find the individual minimum effective dose in both oral and LAI treatments [45,58,59]. Regarding antidepressants, only one study was included in the present review regarding the switch from escitalopram to venlafaxine. It found a positive correlation between venlafaxine blood concentration and adverse effects, such as reduced salivation, orthostatic dizziness, and sweating [60]. All original studies presented about treatment safety are reported in Table 3.

### 3.2. Adherence

Adherence is a multidimensional phenomenon, determined by several factors related to the patient, illness, treatment, and socio-familial milieu. Treatment compliance is a major issue in mental health care, up to 70% of patients have no or only partial adherence to psychopharmacologic treatments [63,64,65]. Poor adherence to treatments is among the few modifiable risk factors for relapse [66], and its monitoring is therefore of crucial importance. Focusing on patients with clinical relapse presenting to emergency services, two studies found a very high prevalence of low drug plasma concentration and estimated that partial adherence or non-adherence could be hypothesized in about two-thirds of patients with schizophrenia and 50% of patients with affective disorders [67,68]. Comparable percentages were observed in a large retrospective study on psychiatric inpatients [69]. In similar samples of people acceding to inpatient emergency facilities because of a relapse of psychotic symptoms, no significant relationship has been found between subjective patient or clinician-rated adherence assessment and drug blood levels [70,71]. In patients with schizophrenia, younger age, poor clinical insight, cannabis, and substances abuse, poor premorbid functioning, the presence of specific symptoms such as paranoic thought, excitement, and lack of impulse control, and polytherapy are associated with low adherence, while the patient and familiar’s positive attitude towards drugs, family involvement, and good illness insight predict adequate compliance [72,73].

Concerning outpatients, a study conducted on a very large sample observed a generally very good level of compliance, with less than 4% of the sample resulting in not adherent [74]. Higher complete non-adherence rates were found in patients receiving olanzapine compared to those receiving other antipsychotics [74]. Since both self-rated and clinician-rated adherence evaluations appear to be unreliable [70,71], TDM can be considered an essential tool in addressing adherence issues, and its use in monitoring compliance to treatment is specifically indicated by AGNP guidelines [2]. In conclusion, TDM stands as a very useful tool in distinguishing between resistance and pseudo-resistance; a larger implementation in routine care, especially in emergency services, where adherence is particularly low, can be of great clinical value. Original articles on TDM application for adherence assessment are presented in Table 4.

### 3.3. Special Populations

Drug absorption, metabolism, distribution, and excretion are subject to relevant variation in individuals with particular conditions, such as pregnancy, limited ages, low or high body weight, and medical or surgical comorbidities. In these populations, the relationships between drug dose, blood concentration, and clinical efficacy are often highly unpredictable.

#### 3.3.1. Peripartum

In several mental disorders, pregnancy and post-partum represent particularly critical phases for biological, psychological, and social factors and are often associated with an increased risk of relapse [75]. Pregnancy implicates alterations in pharmacokinetics, especially in drug distribution, metabolism, and excretion, leading to possibly altered blood concentration and the subsequent impact on clinical efficacy and safety. Closer clinical monitoring is needed, and TDM can play an important role, allowing a more precise dose-finding for the mother ensuring treatment efficacy and safety, and, at the same time, minimizing risks for the fetus or infant related to drug exposure via placenta or maternal milk. AGNP guidelines include pregnancy and breastfeeding as specific indications for TDM for all psychiatric drugs, with the recommendation of performing blood level testing at least once per trimester and shortly after childbirth [2]. The joint consensus statement of the American Society of Clinical Psychopharmacology and AGNP confirms such indication for antipsychotics [76].

Novel data issued in or after 2017 showed a relevant decrease over the pregnancy of serum concentrations of some first- and second-generation antipsychotics, lithium, and variations in several antidepressants’ blood levels, as summarized in the following paragraphs. Focusing on antipsychotics, a large retrospective study on 110 pregnant women receiving antipsychotics revealed a relevant decrease in quetiapine and aripiprazole, up to values below 50% in the third trimester compared with the beginning of pregnancy. Increased CYP3A4 activity may explain an augmented metabolism of quetiapine, while higher CYP2D6 and CYP3A4 expression appears to implicate an increased metabolism of aripiprazole and of its active metabolite dehydroaripiprazole [12,77]. For first-generation antipsychotics, patients treated with perphenazine and haloperidol showed a trend of decreased serum concentration during pregnancy. No significant alterations were identified for olanzapine, while data on other antipsychotics were insufficient to draw relevant conclusions [78]. Focusing on LAI antipsychotic formulations, extremely low paliperidone concentrations in pregnant patients receiving 1-month paliperidone palmitate [11].

Regarding mood stabilizers, significantly lower lithium serum concentrations were observed during the third trimester compared with baseline values [79,80]. Regarding antidepressants, a systematic review and meta-analysis conducted by Schoretsanitis and colleagues searched the literature for mirror studies of comparison between drug blood levels in pregnancy and non-pregnant state in the same patient, highlighting alterations of serum concentration associated with pregnancy for the majority of drugs for which available data were found [81]. Namely, trimipramine, clomipramine, imipramine, nortriptyline, fluvoxamine, citalopram, and paroxetine showed a decrease in dose-adjusted levels, especially in the third trimester, increased concentrations were found for sertraline, whereas no significant alterations in the predicted serum concentration of fluoxetine, escitalopram and venlafaxine were detected [81]. Similar results, albeit with minor differences, were found in a recent transdiagnostic observational study of 60 pregnant women [77]. Moreover, findings from a randomized clinical trial including nine pregnant women treated with sertraline and tested for drug blood levels during the second and third trimesters, showed high interindividual variability in maternal serum concentration, stressing the need for TDM to increase treatment efficacy and safety [81]. A prospective study on breast milk concentration of three antidepressants (citalopram, sertraline, and venlafaxine) revealed that generally only a very low amount of antidepressant drug, in terms of absolute infant dose, was transmitted from mother to child, with the highest values for venlafaxine [82]. This is in line with the observational study of Leutritz et al. 2022 [77].

In conclusion, these results confirm the indication for TDM during pregnancy—especially in the third trimester—in the post-partum weeks, and in the breastfed infants, together with close clinical monitoring of psychiatric symptoms and adverse effects, in order to warrant treatment efficacy and safety of mother and child [12,79,81,82,83,84]. However, in the light of the retrieved literature in this review, as well as of studies presented in previous systematic reviews, available evidence on TDM in pregnancy and post-partum suffers from major limitations: high-quality studies based on large clinical samples are lacking on most drugs and adherence issues, drug–drug interactions and pharmacokinetics factors are often insufficiently addressed. Original articles on TDM use in peripartum considered in our review are listed in Table 5.

#### 3.3.2. Adolescents

Adolescence is a developmental period characterized by major changes in both pharmacokinetics parameters (such as body weight and height) and central nervous system development. Several major psychiatric disorders have onset in adolescence, and psychotropic drugs are largely prescribed in this population [85,86]. However, pharmacotherapy is often associated with suboptimal treatment effects, and evidence in this population is relatively lacking [87]. Moreover, comorbidity with substance use is very common, leading to possible influence on drug metabolism and substance-drug interactions, and adherence issues are a rule more than an exception. Therefore, the dosing regimen and correlations between dose, blood concentration, clinical effects, and toxicity evidenced in adults may not be applicable to adolescents for many psychoactive drugs [88,89,90]. For these above-mentioned reasons, AGNP guidelines strongly recommend TDM to optimize drug treatment in adolescents suffering from mental disorders [2,76]. However, TDM is still largely unemployed in routine clinical practice. Amongst the reasons for this insufficient application of TDM, it is necessary to consider a substantial lack of clear and generally accepted age-specific data assessing the relationship between dose, drug blood levels, and clinical outcomes and defining specific therapeutic and dose-related reference ranges. In reviewing literature published after AGNP guidelines, we considered studies presenting data on patients aged from 14 on.

Only a recent prospective study focused on the relationship between daily dose, serum concentration, and treatment efficacy in adolescents. In particular, it demonstrated higher sertraline daily doses and serum concentrations are more effective in the treatment of OCD in adolescents [91]. Regarding dose-related concentration, about half of the psychotropic drugs assessed in the review by Fekete et al. [92] resulted in having different values compared to adults. Specifically, haloperidol, olanzapine, citalopram, clomipramine, fluvoxamine, and imipramine showed higher dose-related concentrations, while quetiapine, lamotrigine, oxcarbazepine, and topiramate showed lower levels compared to adults. For clozapine, risperidone, ziprasidone, aripiprazole, fluoxetine, paroxetine, sertraline, escitalopram, duloxetine, lithium, valproic acid, carbamazepine, methylphenidate, atomoxetine, guanfacine dose-concentration parameters similar to adults were observed. According to the review conducted by Kloosterboer et al. [93] in an effort to define also for adolescent patients clear blood levels/efficacy/tolerability relationships, an association between drug concentration and both efficacy and side effects was found for methylphenidate and imipramine, a concentration–efficacy relationship was evidenced for quetiapine, citalopram, fluoxetine, nortriptyline, bupropion, and lithium, while a concentration- side effects relationship was highlighted for ziprasidone, venlafaxine, and desipramine. A third recent review specifically addressed selective serotonin reuptake inhibitors (SSRI) TDM in children and adolescents, considering pharmacogenetics profiles, adherence issues, DDI, and drug–substance interactions. Adolescents with low CYP2C19 activity showed higher escitalopram and sertraline exposure and Cmax, while paroxetine clearance resulted in being highly dependent on CYP2D6 activity also for young patients. CBD and THC inhibit CYP activity, and their chronic assumption can therefore lead to increased SSRI plasma concentrations. Oral contraceptives’ effect on reducing citalopram and escitalopram serum concentrations has been observed, whereas proton-pump inhibitors can increase SSRIs blood levels [94].

The three retrospective observational studies, subsequent to the cited reviews, integrate some of the previously lacking information. In particular, Fekete et al. found lower dose-corrected serum concentrations for risperidone and venlafaxine in adolescents compared to adults [95]. The influence of sex and body weight on risperidone and aripiprazole blood levels were analyzed, finding for both drugs higher dose-adjusted concentrations in girls, lower risperidone active moiety serum concentration in lower weight patients and a positive correlation between weight and serum levels of aripiprazole [96,97]. A correlation between higher aripiprazole dose-related serum levels in adolescents and African ethnicity has been hypothesized based on preliminary data [98].

We found two systematic reviews that addressed the issue of TRR and DRRR in adolescence, proposing some novel parameters for various drugs [92,93]. Overall, these two systematic reviews found highly heterogeneous data. Finally, a recent prospective study highlighted an association between higher risperidone serum concentrations and extrapyramidal side effects, suggesting a lower TRR for this antipsychotic in adolescence [99]. In conclusion, adequate studies are lacking for several largely employed drugs in adolescents, most retrieved studies were not of sufficient quality, and most findings were not replicated. Therefore, as already hoped for in the AGNP guidelines [2], in order to better support routine TDM application in adolescents, further high-quality research on the determination of therapeutic and dose-related reference ranges, pharmacogenetic variants, substances, and drug interactions is needed to confirm and integrate present evidence.

#### 3.3.3. Elderly Patients

Aging determines a progressive involvement of the functioning of multiple organs, with a reduction of renal and liver function, and often implies changes in weight and distribution volume. Notably, as most pharmacological clinical trials do not include patients over 65, evidence on this population is limited and based on post-marketing data. Moreover, off-label prescription of psychoactive drugs is relatively frequent. Elderly patients frequently bear the burden of physical comorbidities, are treated with polypharmacotherapy, and often display higher sensitivity to drug adverse effects. About psychotropic drugs, those with anticholinergic activity may particularly hit on elderly patients’ frailty, determining an increased risk of delirium and a decrease in cognitive functions. Considering such elements, TDM in elderly patients is recommended, although evidence on specific reference ranges is still insufficient [2,76].

After the issue of AGNP guidelines, this topic has been reprised by two retrospective analyses using the same large dataset that revealed a significant impact of age on antipsychotic blood levels. In detail, Castberg et al. observed a large effect of age on clozapine, olanzapine, risperidone, and quetiapine dose-adjusted concentration, with a particularly relevant increase from 80 years of age onwards [99]. This effect was most prominent for clozapine, with a 2-fold dose-adjusted concentration for patients aged 80 and a 3-fold dose-adjusted concentration for patients aged 90 compared to patients aged 40 years. Patients treated with olanzapine, the drug for which age-related concentration increase was less prominent, had a 28% dose-adjusted concentration increase at 80 years and a 2-fold increase at 90 years. Concentrations observed in females were generally higher than in males. Jönsson and colleagues conducted analyses on the same large dataset considering patients treated with amisulpride, aripiprazole, clozapine, flupenthixol, haloperidol, olanzapine, perphenazine, quetiapine, risperidone, sertindole, zuclopenthixol, and ziprasidone [100]. For all drugs except flupenthixol and ziprasidone, higher dose-related concentrations were observed in patients over 65 compared to those under 65, with relatively lower concentration/dose ratios for higher drug doses compared to patients receiving low drug doses for most drugs. This study confirmed the finding that in the elderly dose-adjusted concentrations are higher in females than in males.

Subsequent studies confirmed the findings of these two large retrospective analyses for oral administration of amisulpride, zuclopenthixol, olanzapine, and risperidone. Huang et al., in a study conducted on a Chinese population receiving amisulpride, observed an age-related increase in dose-adjusted concentration, leading to a possible indication of a specific age-related dosage of amisulpride (i.e., doses over 600 mg/d should not be recommended in patients over 60) [78]. Another small clinical study on eight patients of different ethnicities suggested relevant receptor occupancy and adequate clinical efficacy at relatively low amisulpride dosage in the elderly [101]. A large study on zuclopenthixol showed an age-dependent serum level increase [102]. For olanzapine, a large sample study by Tveito et al. and two studies on the Chinese population confirmed higher olanzapine dose-adjusted blood concentration and lower concentration of its metabolite desmethyl-olanzapine in the elderly [103,104,105]. Age and sex-related alterations for risperidone were replicated by Fekete et al., with higher dose-corrected serum concentrations of risperidone found in older patients and females [95]. With regard to LAI treatment in the elderly, interesting data showed no age effect on olanzapine LAI absorption and blood levels, in contrast with the olanzapine serum concentration increase observed for the oral formulation while a significant age-dependent increase, especially in women, was observed PP1M and zuclopenthixol, confirming data on the oral formulation, while only small effects of age emerged for fluphenazine and aripiprazole [11,35,102,103,106].

The newest studies on mood stabilizers confirmed linear age-dependent pharmacokinetics for lamotrigine, with up to 30% reduced clearance in the elderly [13,107]. Focusing on antidepressants, sertraline, citalopram, escitalopram, mirtazapine, and venlafaxine blood levels appear to be particularly influenced by age and sex, with a significant proportion of patients in real-world populations, especially elderly women, exposed to concentrations above the TRR [13,45,48,95]. Finally, focusing on the adverse effects of antidepressants in the elderly, a study demonstrated a correlation between amitriptyline and venlafaxine serum concentration and the extent of drug-induced QT prolongation [108]. Based on the studies included in this review [11,13,45,48,78,95,99,100,101,102,103,104,105,106,107,108], TDM may play an important role in optimizing treatment in aged patients, keeping in mind that the elderly, according to the reported data, should not be considered a homogenous population, with age and sex largely influencing drug blood levels. Table 6 presents original articles on TDM in adolescents and the elderly included in our review.

#### 3.3.4. Extreme Body Weight

Weight can have a significant impact on pharmacokinetics, determining alterations, particularly in volume distribution and excretion. However, body weight impact on psychotropic drugs serum concentration is variable and often not of clinically valuable significance; therefore, no on-label specific indication for weight-adjusted dosage is reported for most drugs. Nevertheless, body weight can represent a relevant cause of scarcely predictable concentration/dose ratio, both at the population or individual levels, and abnormal weight is therefore comprised in the AGNP guidelines recommendations for TDM [2].

##### Obesity

A recent study by An et al. conducted on overweight patients receiving olanzapine showed that body weight significantly influences serum concentration, volume distribution, and elimination rate in the Chinese population [105]. Similarly, high BMI can relevantly impact clozapine serum concentration through the augmentation of volume distribution and hepatic enzyme activity alterations, finally resulting in a decrease in clozapine clearance and more elevated plasma levels and concentration/dose ratio [53,110]. Conversely, the concentration/dose (C/D) ratio observed in obese patients receiving PP1M was lower compared to the whole sample [35]. Recently, a clinical study on a real-world population of patients with mania has confirmed the role of body weight in influencing sodium valproate serum concentrations, with an augmented clearance in overweight patients [111]. Similarly, a negative correlation between weight and venlafaxine C/D ratio and between BMI and venlafaxine serum concentrations has been observed [112,113]. Overall, these studies confirm the findings on which guideline recommendations are based, stressing the importance of TDM in overweight patients and over-significant weight variation in the same patient.

##### Low Body Weight and Eating Disorders

Low BMI and/or the presence of an eating disorder can determine highly relevant alterations in drug absorption, distribution, and metabolism. Although abnormally low body weight represents a specific indication for TDM, and its application in eating disorders comprising extremely low body weight patients is potentially beneficial, relevant literature evidence is lacking. No specific body-normalized reference ranges are available for underweight patients, and pharmacologic treatment is mostly empirically conducted [2,113]. Besides a plea for new clinical studies on TDM in low-weight patients and in eating disorders, we found only one work on this issue [112]. It included patients with BMI below 20 kg/m^2^ and observed that sex, and not BMI or weight, could relevantly influence venlafaxine metabolism in the subgroup of low-weight patients, determining higher C/D levels of venlafaxine and its active moiety [112]. Original studies on the role of TDM in patients with abnormal weight are listed in Table 7.

#### 3.3.5. Other Medical and Surgical Conditions That Might Influence Pharmacokinetics

Systemic diseases, especially those involving gastrointestinal, renal, and hepatic systems, can significantly modify most pharmacokinetics parameters. Although renal and liver impairments are probably the most common clinical conditions that impact drug exposure, we found only one relevant review subsequent to the AGNP guidelines issue. It focuses on psychonephrology, i.e., the discipline that studies the connection between mental and kidney health, and suggests that TDM may be a useful tool for adjusting the dosage of psychotropic drugs appropriately in patients with renal disease and also in patients undergoing dialysis [114]. Therefore, we focused on systemic inflammation, HIV infection, and post-surgical conditions, which are the topics about which recent studies provided new significant insights.

Systemic diseases characterized by acute or chronic inflammation may significantly impact drug pharmacokinetics parameters. This effect is mediated mainly through the induction of the acute phase protein α1-acid glycoprotein and variation of other circulating proteins, with consequently altered drug-binding capacity, and via the inhibition of CYP enzyme synthesis mediated by inflammatory mediators such as interleukins 6 and 1, and tumor necrosis factor α. In particular, the interrelationship between inflammation and psychosis is a very intriguing and debated topic. On the one hand, infections and immune system alterations have been implicated in the etiopathogenesis and course of schizophrenia; on the other hand, schizophrenia itself appears to possibly have an influence on the inflammatory status and immune system, with some biological parameters linked to the immune functions prospectively representing possible biomarkers of psychotic disease activity. Antipsychotics also have an impact on the immune system, their therapeutic effect could be partially mediated by immunomodulation, and some of their adverse effects are associated with immune system alterations (metabolic syndrome, myocarditis, and pericarditis) [115].

In the presence of systemic disease, on the one hand, a reduced CYP1A2 activity may increase clozapine blood levels and lead to potentially life-threatening toxicity, while on the other hand, inflammatory status with a marked increase in α1-acid glycoprotein and consequent reduction of unbound drug fraction may minimize adverse effects even in the presence of high clozapine serum concentrations. This complex interaction between inflammation and clozapine distribution and metabolism clinically results in an unstable equilibrium that can determine the occurrence of very high blood levels in the absence of relevant related symptoms [115]. This is in line with the results of our research that found two clinical studies on psychiatric patients with systemic infection showing possible clearance reduction and increased serum concentration of risperidone, clozapine, quetiapine, and aripiprazole [116,117,118]. Considering the above, measurement of individual drug levels over an episode of systemic inflammation, along with closer clinical monitoring of adverse effects, is highly recommended.

Focusing on HIV infection, we found an observational study conducted by Courlet et al. in HIV+ patients treated with escitalopram. It showed that over half of the patients might have serum concentration under the TRR, highlighting relevant and scarcely predictable interindividual variability, with a general tendency towards drug under-exposure, in which drug–drug interaction appears to play a minimal role [119]. Finally, gastrointestinal surgery leads to largely unpredictable alterations in drug absorption, along with subsequent possible body weight decreases and drug distribution volume variations. AGNP guidelines recommend TDM in patients with gastrointestinal resection or bariatric surgery [2]. This indication is confirmed by two studies included in the current review [120,121]. In particular, the observational study by Wallerstedt and Colleagues on patients undergoing bariatric surgery demonstrated high inter-individual variability in drug blood levels, with lower dose-adjusted serum concentrations of sertraline, mirtazapine, duloxetine, and citalopram found after the intervention compared to pre-operatory values [120]. The other work reported two cases of psychiatric patients subjected to bariatric surgery [120]. It evidenced significant antipsychotic serum concentration variations after surgery and suggested a role for long-acting antipsychotic treatment in this population [121]. Table 8 shows original articles on TDM use in patients with systemic inflammation or other relevant medical conditions.

### 3.4. Interactions with Psychotropic Drugs

#### 3.4.1. Drug–Drug Interactions

In the real world, patients often receive more than one drug because they present comorbid medical diseases or need psychotropic polypharmacotherapy. In both cases, potentially relevant drug–drug interactions (DDI) may occur, especially when inhibitors or inducers of drug-metabolizing enzymes are combined with compounds that are the substrate of inhibited or induced enzymes. In these cases, TDM should support individual dosing to ensure treatment efficacy and tolerability. Significantly, in a real-world study on highly adherent patients in polypharmacotherapy, a very high rate of drugs under exposition was found, with almost half of patients having psychotropic drug levels under the therapeutic reference range. Once poor adherence risk is ruled out, polypharmacotherapy stands as a very relevant factor in determining drug exposure, with a need for close clinical monitoring and drug blood level assessment through TDM [122]. Moreover, even when a clear relationship between the coadministration of enzyme inducers and monitored drug’s blood levels are known, as, in the case of lamotrigine, real-world data show that dose adjustments and TDM recommendations are rarely followed [123]. A systematic treatise of drug–drug interactions (DDI) on psychotropic treatments is already presented in previous studies [2,11,13,14,15] and goes beyond the scope of this article. However, we will synthetically focus on the latest evidence, providing an update to the above-cited works.

TDM studies on antipsychotics revealed significant DDI between risperidone and perazine, levomepromazine, melperone, and pipamperone [124,125]. Valproate comedication resulted in decreased olanzapine concentration of up to 40% when adjusting by dose and weight [104]. Coadministration of aripiprazole and other antipsychotics (clozapine, risperidone, quetiapine, and olanzapine) resulted in lower aripiprazole concentration/dose ratios [126]. In patients receiving valproic acid and clozapine, a significant impact on clozapine metabolism was observed, with reduced absolute and dose-adjusted norclozapine serum concentrations [127,128]. Conversely, no relevant effect of sertraline nor pantoprazole comedication was observed on clozapine metabolism [93,94]. Finally, a recent observational study demonstrated a negative relationship between vitamin D levels and dose-adjusted antipsychotic drug concentrations, particularly pronounced for drugs predominantly metabolized by the CYP3A4 (e.g., aripiprazole and quetiapine) [129]. This seems to be due to vitamin D-mediated induction of CYP3A4 [129].

Regarding antidepressants, a significant interaction between sertraline and metamizole was identified, resulting in a decrease in sertraline levels [130]. Venlafaxine metabolism appears to be reduced by quetiapine, frequently employed as augmentation therapy in depression, with higher O-desmethylvenlafaxine and venlafaxine active moiety levels observed [131]. Omeprazole and pantoprazole also showed an inhibitory effect on venlafaxine metabolism [132]. A prospective study on fluoxetine, assessing both drug concentrations and clinical response, revealed an association between depressive symptoms and the presence of CYP2D6 inhibition due to a co-administered drug [133]. We found some studies that demonstrated specific DDIs [124,125,126,127,128,129,130,131,132,133,134,135]; however, it has to be considered that significant variations of drug blood concentrations observed in most of the reported studies are not necessarily associated with altered clinical efficacy or tolerability, and further research is needed to determine the clinical relevance of observed interactions.

#### 3.4.2. Drug–Smoke Interaction

Although smoking habits are not directly reported in AGNP guidelines and TDM recommendations, smoke can have a large impact on drug metabolism. Smoking directly induces hepatic CYP1A2; therefore, patients receiving drugs that are CYP1A2 substrates may show reduced drug blood levels. To help clinicians’ decision-making, it has to be kept in mind that an average of 10 cigarettes per day is sufficient to reach the maximum of CYP1A2 induction so that variations in smoking habits, such as passing from 5 to 10 cigarettes a day or vice versa can be of clinical relevance, whereas changes from 10 to 20 or more do not imply significant pharmacokinetic changes. Moreover, electronic cigarettes or nicotine patches do not have the same impact on CYP1AP activity, and therefore, changes in patients’ habits from smoking to these products need to be considered. Other ways through which smoke can affect drug metabolism and effects have also been hypothesized, such as the effect of nicotine in altering brain-blood barrier permeability to drugs and epigenetic and microbiome modifications [115]. An indirect relationship between smoke and low drug serum concentration may also be present in psychiatric patients, as smoking is significantly related to lower cognitive performance, global functioning, economic status, and poorer prognosis [136,137,138]. These clinical and demographic characteristics may be associated with lower illness insight and awareness of the need for treatment and consequent poor adherence, as well as a higher prevalence of unhealthy lifestyles habits such as alcohol and substance use, with the more frequent presence of comorbidities and co-medications and possible drug–drug or drug–substance interactions [137,139]. This may finally result in lower serum concentrations of psychopharmacological treatments in smokers, as confirmed by a recent real-world study in an emergency setting [72].

The role of CYP1A2 induction is well studied for several drugs, i.e., olanzapine and clozapine, and is reported in the review cited above [115]. Nevertheless, it is worth signaling some more recent work confirming clinically relevant interactions between smoke and drug plasma levels that were not previously adequately verified in clinical samples [140,141,142,143]. Considering the direct and indirect relationships between smoke and the finding of lower drug serum concentrations found in smokers compared to non-smokers, TDM can be a very useful clinical tool for this very frequent sub-population of people with mental disorders. Studies conducted in naturalistic settings confirmed the role of smoke in reducing duloxetine levels via induction of CYP1A2 [109], found no smoke impact on paliperidone metabolism, and proved a beneficial effect of fluvoxamine augmentation in increasing clozapine blood levels in smokers, neutralizing smoke effect on CYP1A2 activity [141,142]. Finally, a retrospective naturalistic study observed reduced amitriptyline and mirtazapine serum concentrations in smokers, although CYP1A2 does not theoretically play a major role in their metabolism, enlarging the possible role of CYP2C19 e CYP3A4 induction. On the other hand, no differences between smokers and non-smokers were detected for risperidone, about which CYP3A4 induction role in reducing its concentrations is still debated [143].

#### 3.4.3. Other Interactions

Drugs and smoke are obviously not the only factors involved in pharmacokinetic interactions. Food and alcohol intake may significantly impact drug absorption and clearance. Clinicians should be aware of food and alcohol’s role in influencing drug concentrations, and food and alcohol intake habits need to be expressively addressed in clinical interviews. Food may alter drug availability influencing absorption, as in the case of lurasidone and ziprasidone, whose absorption is increased by fatty food and through specific CYP induction or inhibition [2]. Similarly, alcohol use effects on drug metabolism may be mediated by CYP2E1 induction or indirectly via the chronic liver or gastric alterations [2]. About this topic, we found one retrospective observational study on the use of TDM for patients with major depressive disorder and alcohol use disorder treated with escitalopram [144]. According to this study, alcohol dependence alone does not lead to pharmacokinetic changes in the metabolism of escitalopram but altered liver function, in terms of elevated GGT in combination with an AST/ALT ratio ≥ 1, does [144].

### 3.5. Pharmacogenetics and TDM

A systematic treatise of the topic would require a specific focus that goes beyond the scope of this review. However, we provide a rapid overview of what is new after AGNP guidelines on the combined use of TDM and pharmacogenetics testing.

#### 3.5.1. Antipsychotics

A significant impact of CYP2D6 variants, namely nonfunctional variant alleles (CYP2D6*3, *4, *4N, *5, *6, *7, *8, *11, *12, *13, *14A, *15, *36, *68) and the reduced function variant alleles (CYP2D6 *2, *9, *10, *14B, *17, *29, *41) has been observed for several first and second-generation antipsychotics [101,102,103,104,105]. In particular, a 3.9-fold increase in dose-adjusted serum concentration of perphenazine, a 1.5-fold increase for zuclopenthixol, 1.6-fold for risperidone, 1.4-fold for aripiprazole, and a significant increase for olanzapine in poor metabolizers (PM, i.e., patients with only null alleles) compared with normal metabolizers have been observed [145,146,147,148,149]. Conversely, risperidone treatment failure has been associated with a CYP2D6 variant (CYP*2A, a variant with increased metabolic activity) or multiple copies of CYP*1 or *35 (variants with normal activity) [146,150], while a higher maximum dose of aripiprazole has been proposed for ultrarapid metabolizers (UM, i.e., patients with two CYP2D6*2A alleles or three or more normal activity alleles) [149,150]. Focusing on clozapine treatment failure due to increased metabolism, we found three original contributions that adopted a pharmacogenetic approach. A genome-wide association study revealed a possible role of a non-previously considered polymorphism in modulating clozapine serum levels, namely the rs28379954 T > C polymorphism of the Nuclear Factor IB (NFIB) gene, that links to DNA and favors DNA transcription by RNA polymerase II. NFIB appears to be involved in the transcription of genes involved in clozapine metabolism, leading to reduced clozapine levels [151]. A case report found a significant role of the CYP1A2*1F variant in determining low clozapine level and clinical non-response [152], while another case report of treatment failure with low clozapine blood level detected highlighted the importance of considering clozapine metabolism as dependent on the genetic profile of various CYP genes, such as CYP1A2, CYP3A4, CYP2C19, and CYP2D6, and of UGT1A4 and ABCB1 genes [28].

#### 3.5.2. Antidepressants and Other Psychotropic Drugs

About antidepressants, personalized dosing according to CYP2D6 and CYP2C19 variants has been proposed for several antidepressants and can be retrieved in specific guidelines and reviews [13,14,15,153]. CYP2D6 variants are the same as described in the antipsychotic subsection, while CYP2C19 variants are classified as follows: null activity (CYP2C19*2, *3, *4, *5, *6, *7, *8, *10, *11), decreased activity (CYP2C19*2, *3, *4, *5, *6, *7, *8, *10, *11), increased activity (CYP2C19*17). CYP2C19 PM are defined as people with two null alleles, while CYP2C19 UM as those with two increased activity alleles [149].

Studies on a very large database confirmed the role of both CYP2D6 and CYP2C19 on amitriptyline [154] and venlafaxine metabolism, with up to 13-fold dose-adjusted serum concentration in patients with PM profiles for both CYPs [155], and a very low enzyme function in CYP2D6*41 carriers has been observed [147,156]. A significant impact of CYP2D6 has also been confirmed for fluoxetine, with an increase in fluoxetine serum concentration and low norfluoxetine levels and norfluoxetine/fluoxetine ratio in PM and intermediate metabolizers (IM, i.e., patients with one normal activity allele with one null activity allele or with two decreased activity alleles) [133,149].

Conversely, three recent studies focused on the CYP2C19*17 genetic variant that is associated with the increased metabolic activity of the CYP. In detail, lower serum concentrations of venlafaxine without variations in terms of antidepressant response in patients with one copy of the CYP2C19*17 genetic variant were found by Sherf-Clavel et al., while Zastrozhin and Collaborators reported a negative association between the CYP2C19*17 genetic variant and the antidepressant efficacy and safety of escitalopram treatment without a significant variation in the serum concentration of the drug in these subsample of patients [157,158]. Finally, a case report on a patient with scarce clinical response to bupropion revealed a previously not observed in-vivo role of an alternative metabolic pathway via CYP2C19, with UM status appearing related to low bupropion active moiety levels and low efficacy [159].

With regard to other classes of psychotropic drugs used in psychiatry, we retrieved only a study on depressed patients receiving lamotrigine as augmentation therapy that showed no relevance of UDP-glucuronosyltransferase gene polymorphism and drug plasma concentration and work on a sample of patients with anxiety disorder and alcohol use disorder, highlighting the impact of a specific polymorphism of CYP3A4 gene (99366316G > A) in determining alprazolam higher efficacy and presence of adverse effects [160,161]. In conclusion, the interplay between genetic profile, drug metabolism, C/D ratios, clinical response, and adverse effects is complex and scarcely predictable at the individual level, as it is the result of a complex multifactorial process based on genetic and non-genetic variables which multiple genes involved in drug metabolism, transcellular transport, drug receptors, and intracellular signaling need to be considered. AGNP guidelines recommend TDM in the presence of a genetic peculiarity concerning drug metabolism [2]. This agrees with the results of the studies summarized in which it has been evidenced as TDM and pharmacogenomics combined can provide useful information on the effectiveness and safety of psychoactive drugs [145,146,147,148,149,150,151,152,153,154,155,156,157,158,159,160,161].

### 3.6. Novel Approaches toward Minimally Invasive TDM

A variety of novel minimally invasive techniques receives growing interest as a promising way to increase TDM implementation in clinical practice through procedures with easy access, home or point-of-care availability, and user-friendly devices.

#### 3.6.1. Dried Blood Spot Analysis (DBS)

Among the most studied procedures stands dried blood spot (DBS) analysis, generally carried out through a simple finger prick. Sampling can be performed by the patient himself at home or in points-of-care without the need of facilities usually needed for vena punctures. DBS is more user-friendly compared to vena puncture and can be particularly helpful in improving the elderly’s access to TDM since no displacement to a health care center is required. According to the studies found in our research, DBS appears to be a reliable tool in performing TDM of clozapine, ziprasidone, lamotrigine, valproate, lithium, citalopram, fluoxetine, sertraline, venlafaxine, and vortioxetine [162,163,164,165,166]. Up to date, no clinical validity has been reported for DBS analyses for TDM of risperidone, aripiprazole, and pipamperone, and possible overestimation of clozapine and amitriptyline exposure through DBS TDM compared to traditional TDM has been suggested [163,167]. Overall, DBS has a generally lower sensitivity than traditional TDM based on blood levels and is more subjected to pre-analytical and analytical bias related to the variability of hematocrit values and to possible errors in remote sampling [163,167].

#### 3.6.2. Volumetric Absorptive Microsampling (VAMS)

Novel promising microsampling techniques are emerging, such as VAMS. Its main advantages, in comparison with DBS, are a more precise and accurate collection of blood volumes, minimization of hematocrit effect on results, easier post-analytic sample management, and reduced costs [168]. Recent validation studies suggest that volumetric absorptive microsampling (VAMS) and other microsampling procedures may be reliable and smart techniques for TDM in psychiatry. In particular, blood, plasma, and oral fluid VAMS in patients receiving, sertraline, fluoxetine, citalopram, or vortioxetine provided results in valid agreement with those obtained with routinary methods [165]. Moreover, innovative volumetric absorptive paper discs have been proposed for assessing levels of clozapine and its metabolites, demonstrating good accuracy of the new methods [162,169].

VAMS and other innovative capillary microsampling procedures [166], feasibility, accuracy, and reliability have been proven and clinically validated for several psychotropic drugs [165,166,169,170,171]. VAMS stands today as a very promising mini-invasive TDM procedure in terms of easy applicability and clinical reliability, making precision psychiatry closer. Nevertheless, further studies with adequate clinical validation and a clear assessment of agreement between plasma and DBS/VAMS monitoring methods are needed [163].

#### 3.6.3. Oral Fluid Analysis

The oral fluid analysis represents another promising method toward an easily accessible TDM: the collection is non-invasive, can be carried out at home, and allows simple monitoring in patients for whom blood sampling can be problematic or undesirable. Data indicating the potential utility of saliva TDM are available for clozapine, risperidone, quetiapine, olanzapine, venlafaxine, lithium, valproate, carbamazepine, lamotrigine, and methylphenidate [162,172,173,174]. A single study, on the contrary, found no validity in monitoring amphetamine treatment in children and adolescents with ADHD through saliva collection [175]. In conclusion, available data are still limited and heterogeneous as saliva drug concentrations are generally lower than plasma, with consequent more complex measuring. However, oral fluid analysis emerges as a promising instrument in assessing treatment adherence for several psychotropic drugs comprising antipsychotics and antidepressants [162,172,173,174,176].

#### 3.6.4. Other Non- or Mini-Invasive Procedures

A further strand of TDM development with different methods, biological samples, and intended uses has been the subject of some recent studies included in the current review [177,178,179,180,181,182]. Completely non-invasive wearable devices have been developed for TDM of lithium [177,178], and novel biosensors may constitute a reliable, cost-effective, and point-of-care disposable alternative to current laboratory techniques [180,181]. Moreover, seminal fluid TDM could be helpful in managing reduced fertility in patients receiving antipsychotics or antidepressants [181]; hair testing can be a sensitive method in assessing treatment adherence over a large timeframe, especially for lamotrigine and carbamazepine [85]; and urine metabolites dosage represents a possible tool in assessing compliance to novel antipsychotics treatment [182]. In summary, these are new technologies under development, but they may become the subject of future scientific studies and, consequently, useful tools for clinical practice.

### 3.7. Towards Precision Pharmacotherapy in Psychiatry

Precision medicine is a healthcare approach that considers specific characteristics of patients such as sex, age, genetics, metabolic, environment, lifestyle, etc., and of their diseases (e.g., the genetic profile of a tumor) in order to provide a tailored therapeutic intervention, maximizing treatment efficacy, minimizing toxicity and generally improving the efficiency of healthcare systems. Treatment personalization needs reliable indicators of the development and evolution of the disease and of treatment response, which can consist of measurements of different biological traits through genetic and metabolic analysis or bioimaging. The past decade has been characterized by specific efforts to achieve tailored intervention in psychiatry. A new framework to define mental disorders according to genetic, molecular, and neuronal pathways; physiological variables; and behavioral characteristics has been proposed [183]. Ongoing efforts are produced to achieve satisfying clinical staging models for the most common and severe mental disorders in order to define disease progression, predict prognosis, and allow tailored treatment [184,185]. Large-scale genetic studies brought a growing body of information on the genetic basis of major psychiatric disorders [186,187], and progress in neuroimaging and electrophysiology allows a more exhaustive comprehension of pathological processes and of the role of treatments [188,189]. The large field of precision psychiatry comprises precision psychopharmacotherapy. Several biomarkers have been identified as related to treatment outcomes and safety, namely cytochrome P450s enzymes [190], genes, and proteins involved in DNA transcription and RNA translation or in intracellular signaling and neurotransmission [191,192,193,194]. TDM stands as a tile in this complex mosaic, enabling more precise employment of pharmacotherapy through treatment personalization, especially when performed synergically with genetic profiling, as seen in Section 3.5, in a variety of clinical conditions, such as treatment resistance or pseudo-resistance, unforeseen adverse effects, pregnancy, limit ages, abnormally high or low weight, presence of comorbidities or other pharmacological treatments. Moreover, mini-invasive TDM procedures appear to be a road to follow to extensively bring precision pharmacotherapy into everyday clinical practice.

The principal limitations of this review are the non-systematic nature of the review, which did not allow for accurate screening of the methodological quality of the included records, and the choice to search exclusively the Web of Science platform and no other databases such as PubMed. The main strength of this paper is the breadth of the topics covered that provide a summary of the literature on the use of TDM in psychiatry since the publication of the AGNP guidelines.

## 4. Conclusions

TDM receives growing research and clinical interest; however, it remains underemployed in the real world. Among the reasons for its relatively limited use is a lack of clear correlation between drug serum concentration and efficacy and tolerability, both in the general population and even more in patients with specificities of drug metabolism. Recent evidence, issued after the latest AGNP guidelines, help to shed light on some TDM application field still scarcely explored. In particular, some recent studies demonstrate a correlation between serum drug concentrations at the treatment start and clinical efficacy and safety, especially for antipsychotics. This might help clinicians in making decisions on early laboratory findings, possibly avoiding a trial-and-error approach. A similar result was also obtained for first-line antidepressant treatments. Concerning populations with specific pharmacokinetic characteristics, the studies included in the current review confirmed frequent alterations of serum drug concentrations in pregnant women, generally with a progressive decrease over pregnancy and a relevant dose-adjusted concentration increase in the elderly. As compared to adults, also in adolescents, several drugs result having different dose-related concentrations. Pharmacogenetic analyses combined with TDM may help clinicians in choosing more effective and safe psychopharmacologic treatments, addressing therapy on an individual pharmacometabolic basis. Mini-invasive TDM procedures may be easily performed at home or at a point-of-care level and may enhance the use of TDM on a regular basis for many psychotropic drugs in different real-world settings.

Although the recent evidence summarized in this review, research efforts have to be carried on: further studies are needed to replicate present findings and provide clearer knowledge on relationships between dose, serum concentration, and efficacy/safety. To accomplish this, prospective fixed-dose studies in the general population and in specific populations with distinctive pharmacokinetic characteristics are needed. Furthermore, a larger sample size of studies is required to obtain more accurate scientific evidence with a good balance of statistical power and significance. These goals could be achieved, for example, through consortia aimed at designing multicenter studies with a shared methodology designed to expand knowledge about TDM in psychopharmacology. In conclusion, an investment of economic resources aimed at conducting this kind of studies and an effort to diffuse the knowledge on this topic in order to train psychiatrists on the proper use of TDM in a wider range of real-world contexts would allow clinicians and patients to benefit from this precious technique.

## Figures and Tables

**Table 1:** TDM recommendation in neuropsychopharmacological treatment.

Obligatory TDM
Dose optimization after initial prescription or after dose change for drugs with a high level of recommendation to use TDM
Drugs for which TDM is mandatory for safety reasons (e.g., lithium or carbamazepine)
Specific indications for TDM
Uncertain adherence to medication
Relapse prevention because of uncertain adherence to medication
Lack of clinical improvement under recommended doses
Relapse under maintenance treatment
Determination of optimal individual drug concentration when the patient has attained the desired clinical outcome
Recurrence of symptoms under adequate doses
Clinical improvement and adverse effects under recommended doses
Combination treatment with a drug known for its interaction potential or suspected drug interaction
Use of counterfeit medications by the patient
Presence of a genetic peculiarity concerning drug metabolism (genetic deficiency, gene multiplication)
Patients with differential ethnicity
Patients with abnormally high or low body weight
Pregnant or breastfeeding patient
Children or adolescent patient
Elderly patient (>65 y)
Patients with intellectual disability
Patients with pharmacokinetically relevant comorbidity (hepatic or renal insufficiency, cardiovascular disease)
Patients with acute or chronic inflammations or infections
Patients with restrictive gastrointestinal resection or bariatric surgery
Problems occurring after switching from an original preparation to a generic form (and vice versa)
Use of over-the-counter (OTC) drugs by the patient

TDM: therapeutic drug monitoring.

**Table 2:** Original articles on the use of TDM to ensure treatment efficacy.

Study	Study Design	Drugs	n	Age	Sample Characteristics	Diagnosis	Outcome
Arnaiz et al., 2021 [18]	Prospective multicentric	Olanzapine	47	26.2 ± 5.1 (mean ± SD)	Patients with FEP; ethnicity: Caucasian, other	Affective and non-affective first psychotic symptoms of at least 1-week duration	Positive association between drug C/D ratio and clinical response
Bustillo et al., 2018 [49]	Prospective multicentric	SGA	64	28.7 ± 7.3 (mean ± SD), 18–49 (range)	Patients with FEP; ethnicity: Caucasian, Hispanic	SSD and BD	Drug SC is not associated with early clinical response
Cellini et al., 2022 [46]	Prospective	Escitalopram, Duloxetine, Venlafaxine, Mirtazapine	206	58.11 ± 16.85 (mean ± SD)	Patients with current major depressive episode (HAMD-21 ≥ 14) treated with first-line AD ethnicity: Caucasian, other	MDD	Concentration-dependent clinical efficacy of first-line ADs with a bell-shaped quadratic function indicating a progressive increase in AD efficacy up to around the upper normalized limit of the TRR with a decrease in the AD response at higher SC.
D’Anna et al., 2022 [39]	Prospective	LAI SGA	48	44.33 ± 12.63 (mean ± SD)	Clinically stable outpatients	SSD	Under-range LAI levels predicted relapse in SSDs
Grover et al., 2022 [27]	Cross-sectional	Clozapine	50	35.7, 19–62 (mean, range)	Clozapine responders and non-responders, Asian ethnicity	Treatment-resistant schizophrenia	Association between a higher clozapine/norclozapine ratio and clinical non-response
Hyza et al., 2021 [38]	Retrospective	LAI FGA and SGA	40	45 ± 13 (mean± SD)	At least 3-month LAI treatment, ethnicity not stated	Schizophrenia and related disorders	High prevalence of subtherapeutic drug SC
Kauffmann et al., 2016 (corrigendum 2020) [22]	Prospective	SGA	87	34.7 ± 79.9 (mean ± SD), 18–58 (range)	In- and outpatients starting SGA monotherapy; ethnicity not stated	SSD	No correlation between clinical response and observed drug SC
Kylleso et al., 2021 [25]	Retrospective	Clozapine	190	Switchers: 39 ± 2 (mean ± SD); non-switchers: 43 ± 1 (mean ± SD)	Patients switching from clozapine to other AP	Schizophrenia	Association between clozapine discontinuation and low SC
Mauri et al., 2020 [43]	Prospective	LAI aripiprazole and LAI paliperidone	56	Aripiprazole: 41.92 ± 13.28 (mean ± SD); paliperidone: 40.83 ± 13.32 (mean ± SD)	Patients clinically stabilized with oral treatment¸ ethnicity not stated	BD with manic predominance	Lower paliperidone SC may have positive effect on depressive symptoms
McCutcheon et al., 2018 [32]	Retrospective	FGA and SGA	99	Patient with SC in therapeutic range: 44.4 (median); patient with low SC: 35.7 (median)	Patients with treatment-resistant schizophrenia; ethnicity: White British, Black	Schizophrenia, schizoaffective disorder, other	Association between resistance to treatment and subtherapeutic drug SC
Melkote et al., 2018 [31]	Retrospective analysis of data from prospective RCT	Olanzapine, risperidone	316	40.9 (mean), 43 (median), 18–67(range)	Patient included in CATIE study undergoing treatment failure; ethnicity: Caucasian, African American, other	Schizophrenia	Correlation between treatment failure and antipsychotics SC below TRR
Nagai et al., 2017 [19]	Prospective	Aripiprazole	26	37.7 ± 12.8 (mean ± SD)	Japanese inpatients, early treatment phase	Schizophrenia	Optimal dose predictable through aripiprazole + dehydroaripiprazole TDM at week 1
Olmos et al., 2019 [29]	Prospective	Clozapine	98	39 (median), 20–67 (range)	Caucasian	Schizophrenia	Bioequivalence of two clozapine brands
Oloyede et al., 2020 [30]	Prospective	Clozapine	28	47 ± 11.59 (mean ± SD)	In- and outpatients; ethnicity: Asian, Black, Caucasian	Schizophrenia	Bioequivalence of two clozapine formulations
Paulzen et al., 2017 [23]	Retrospective	Risperidone	590	Responders: 46.1, 18–82 (mean, range); Non-responders: 40.9, 18–87 (mean, range)	Ethnicity not stated	Not stated	Positive association between risperidone active moiety and clinical response
Schoretsanitis et al., 2021 [36]	Retrospective	PP1M	173	Responders: 44.0, 32.0–59.0 (median, IQR); non-responders: 47.5, 39.8–58.5 (median, IQR)	Ethnicity not stated	SSD, BD, other	Possible specific patterns of clinical response according to diagnosis and SC
Schoretsanitis et al., 2022 [37]	Retrospective	PP1M	183	Control: 43.0, 33.0– 62.5 (median, IQR); Overweight: 47.0, 34.2–59.7 (median, IQR); Obese: 49.0, 39.2–57.7 (median, IQR)	Adults, elderly, normal and overweight patients	SSD, BD, other	High interindividual variability of SC, no influence of age, sex, smoking, or body weight on SC
Tien et al., 2022 [20]	Prospective	Aripiprazole	64	Aripiprazole SC: ≤300 ng/mL: 35.2 ± 14.9; >300 ng/mL: 34.3 ± 11.3 (mean ± SD)	Ethnicity; Asian—Taiwan	Schizophrenia	Higher response in terms of Clinical Global Impressions (CGI) scores with aripiprazole SC > 300 ng/mL (higher than the western population-based TRR).
Yada et al., 2021 [24]	Cross-sectional multicentric	Clozapine	131	40.1 ± 12.0 (mean ± SD), 16–72 (range)	Japanese patients	Treatment-resistant schizophrenia	Confirmed validity of AGNP clozapine TRR; doses above 1000 ng/mL are more effective but have higher toxicity risk

AP: antipsychotics; BD: bipolar disorder; C/D: concentration/dose; CATIE: Clinical Antipsychotic Trials of Intervention Effectiveness; FEP: first episode psychosis; FGA: first-generation antipsychotics; LAI: long-acting injectable; MDD: major depressive disorder; PP1M: once-monthly paliperidone palmitate; RCT: randomized clinical trial; SC: serum concentration; SGA: second-generation antipsychotics; SSD: schizophrenia spectrum disorders; TRR: therapeutic reference range.

**Table 3:** Original articles on the use of TDM to ensure treatment safety.

Study	Study Design	Drugs	n	Age	Sample Characteristics	Diagnosis	Outcome
An et al., 2022 [57]	Cross-sectional	Olanzapine	352	56.6 ± 14.2 (mean ± SD)	Inpatients	Schizophrenia	Negative association between plasma DMO concentrations and glucose, insulin, and triglycerides SC Positive association between plasma olanzapine concentrations and C-reactive protein and homocysteine SC
Arnaiz et al., 2021 [18]	Prospective multicentric	Olanzapine	47	26.2 ± 5.1 (mean ± SD)	Patients with FEP; ethnicity: Caucasian (94.2%), other	Affective and non-affective first psychotic symptoms of at least 1-week duration	Positive correlation between drug SC and weight gain
Diaz et al., 2017 [53]	Retrospective analysis of data from RCT	Clozapine	47	45 ± 10 (mean ± SD), 28–62 (range)	Patients included in RCT; ethnicity: African Americans, Caucasians	Schizophrenia	Association between weight gain and higher SC
Engelmann et al., 2021 [60]	RCT	Venlafaxine	234	39.9 ± 12.2 (mean ± SD)	Non-responders to first-line treatment with escitalopram, ethnicity not stated	MDD	No significant correlation between venlafaxine SC and ADRs
Kang et al., 2022 [56]	Prospective	Olanzapine	117	Drug-naïve: 27.50 (24.83–30.17) Chronic: 38.82 (36.19–41.45) (Interquartile range)	First episode drug-naïf and patients with a duration of illness > 5 years ethnicity not stated	Schizophrenia	Metabolic dysfunction is more severe and dose-dependent in drug-naive patients but independent in patients with chronic schizophrenia
Kitchen et al., 2021 [50]	Retrospective	Clozapine	874	-	Patients undergoing routine TDM; ethnicity not stated	Schizophrenia, schizoaffective disorder	Reduction of patients with high-risk clozapine SC through routinary TDM implementation
Lu et al., 2018 [58]	Prospective	Olanzapine	151	41.3 ± 12.1 (mean ± SD)	In- and outpatients	Schizophrenia	Negative correlation between DMO C/D ratio and presence of metabolic syndrome. Positive correlation between olanzapine SC/DMO SC ratio and presence of metabolic syndrome. Proposal of a range for olanzapine SC/DMO SC (3–6) to maximize efficacy and reduce metabolic side effects
Schoretsanitis et al., 2021 [61]	Retrospective	PP1M	172	Patients with ADR: 50.5 (25.0) (median, IQR); patients without ADR: 47.0 (23.2) (median, IQR)	In- and outpatients, ethnicity not stated	Not stated	Paliperidone C/D ratio over 7.7 (ng/mL)/(mg/day) associated with higher ADRs risk
Schoretsanitis et al., 2021 [54]	Retrospective	Clozapine	395	Patients with hypersalivation: 41.5 (21.0) (median, IQR); patients without hypersalivation: 41.0 (22.0) (median, IQR)	In- and outpatients, ethnicity not stated	Not stated	Correlation between high clozapine SC and C/D ratio and hypersalivation
Smith et al., 2017 [52]	Retrospective	Clozapine	129	34, 20–84 (median, range)	Ethnicity not stated	Schizophrenia	Correlation between norclozapine SC and granulocyte count
Veselinović et al., 2019 [62]	Retrospective analysis of data from RCT	Aripiprazole, flupentixol, haloperidol, olanzapine, quetiapine,	69	34.8 ± 10.9 (mean ± SD)	Patients included in RCT; ethnicity not stated	Schizophrenia	Association between high SC, high D2 receptors occupancy, and low subjective well-being
Yada et al., 2021 [24]	Cross-sectional multicentric	Clozapine	131	40.1 ± 12.0 (mean ± SD), 16–72 (range)	Japanese patients	Treatment-resistant schizophrenia	Confirmed validity of AGNP clozapine TRR; doses above 1000 ng/mL are more effective but have higher toxicity risk

AD: antidepressant; ADR: adverse drug reaction; C/D: concentration/dose; DMO: N-desmethyl-olanzapine; HAMD-21: Hamilton Depression Rating Scale-21 items; MDD: major depressive disorder; PP1M: once-monthly paliperidone palmitate; RCT: randomized clinical trial; SC: serum concentration; TRR: therapeutic reference range.

**Table 4:** Original articles on the use of TDM to ensure treatment adherence.

Study	Study Design	Drugs	n	Age	Sample Characteristics	Diagnosis	Outcome
Baldelli et al., 2020 [69]	Retrospective	AP, AD	1197	Not stated	Italian database; ethnicity not stated	Not stated	45% of patients with SC below TRR
Brasso et al., 2021 [71]	Prospective	AP, mood stabilizers	133	Patients with SSD: 43.1 ± 13.5 (mean ± SD); patients with BD: 51.9 ± 14.4 (mean ± SD)	Inpatients in a psychiatric emergency service; ethnicity: Caucasian, other	SSD, BD, and related disorders	50% of patients with SC out of TRR; no correlation between TDM and self-assessment of adherence
Geretsegger et al., 2019 [67]	Prospective	AP, AD	161	40.4 (mean)	Inpatients newly admitted to a psychiatric emergency service; ethnicity not stated	Psychotic disorders, mood disorders, other disorders	52% of patients with SC below TRR, patients with psychotic disorder are less adherent compared to patients with mood disorders
Lopez et al., 2017 [70]	Prospective	Aripiprazole, risperidone, olanzapine, paliperidone, quetiapine	97	39, 18–74 (mean, range)	Patients acceding to a psychiatric emergency service; ethnicity: African American, Asian, White, Hispanic, other	Schizophrenia, schizoaffective disorder, BD, psychotic disorder not otherwise specified	66% of patients with SC out of TRR; no correlation between TDM and clinician assessment of adherence
Silhan et al., 2018 [68]	Prospective	Citalopram, escitalopram, paroxetine, sertraline, venlafaxine	83	40.3 ± 12.2 (mean ± SD)	In- and outpatients; ethnicity not stated	Depressive disorders, anxiety disorders	37.3% of non-adherent patients in the whole sample; 56.8% of the outpatient subgroup with SC below TRR
Smith et al., 2020 [72]	Retrospective	AP	24,239	44 (median), 15–106 (range)	Norwegian database; ethnicity not specified	Not stated	AP polypharmacy is associated with non-adherence
Smith et al., 2021 [74]	Retrospective	Aripiprazole, clozapine, olanzapine, quetiapine, risperidone	13,217	44.3 ± 16.0 (mean ± SD)	Norwegian database, outpatients; ethnicity not specified	Schizophrenia, other	Complete non-adherence in less than 5% of patients with psychotic symptoms; higher non-adherence in patients receiving olanzapine compared to other drugs

AD: antidepressants; AP: antipsychotics; BD: bipolar disorder; SC: serum concentration; SSD: schizophrenia spectrum disorders; TRR: therapeutic reference range.

**Table 5:** Original articles on the use of TDM during peripartum.

Study	Study Design	Drugs	n	Age	Sample Characteristics	Diagnosis	Outcome
Clark et al., 2022 [80]	Prospective	Lithium	3	22–39 (range)	Women during pregnancy and post-partum; 1 Caucasian, 1 Hispanic, 1 Afro-American	BD, type I	Lithium elimination clearance increase of 63.5% by the third trimester; lithium elimination clearance was inversely related to SC; mood symptoms worsened with declines in SC; lithium elimination clearance returned to baseline at 4 to 9 weeks postpartum
Heinonen et al., 2021 [83]	RCT	Sertraline	9	24–39 (range)	Women during pregnancy and post-partum, infants; ethnicity not stated	Depression	High interindividual maternal serum concentration, low infant exposition
Leutritz et al., 2022 [77]	Retrospective and Prospective	Amitriptyline, Aripiprazole, Bupropion, Citalopram Clomipramine, Duloxetine, Escitalopram, Lamotrigine, Mirtazapine Paroxetine, Quetiapine, Sertraline, Venlafaxine	60	33.26 ± 2.45 (mean ± SD)	Outpatients during pregnancy and post-partum infants; ethnicity not stated	Major depressive disorder, Anxiety disorders, Obsessive–compulsive disorder, Bipolar disorders, Schizoaffective disorder, Adjustment disorder, Substance use disorders	↓ SC from the I to the II trimester of amitriptyline, duloxetine, escitalopram, quetiapine, and sertraline; citalopram stayed stable during pregnancy, ↑ sertraline SC from the II to the III trimester; high concentration-by-dose ratios in breastmilk for venlafaxine and lamotrigine, low for quetiapine and clomipramine
Schoretsanitis et al., 2019 [82]	Prospective	Citalopram, sertraline, venlafaxine	17	23–40 (range)	Breast-feeding women; ethnicity not stated	Depressive episode	Higher breastfed children exposure to venlafaxine
Westin et al., 2017 [79]	Retrospective	Lithium	13	32.9 ± 3.8 (mean ± SD)	Pregnant women, assessment at baseline, during pregnancy, and after delivery; ethnicity not stated	BD	↓ lithium SC during III trimester
Westin et al., 2018 [84]	Retrospective	AP	103	29 (mean)	Pregnant women, assessment at baseline and during pregnancy; ethnicity not stated	Not stated	↓quetiapine and aripiprazole SC during III trimester

AP: antipsychotics; BD: bipolar disorder; RCT: randomized clinical trial; SC: serum concentration.

**Table 6:** Original articles on the use of TDM in adolescents and elderly patients.

	Study	Study Design	Drugs	n	Age	Sample Characteristics	Diagnosis	Outcome
Adolescents	Egberts et al., 2020 [97]	Retrospective	Aripiprazole	130	15.0 ± 2.6 (mean ± SD), 7.6–19.0 (range)	65% of the patients treated with polytherapy; ethnicity not stated	SSD, mood disorders, other	Aripiprazole TRR for adolescents similar to adults; positive correlation between weight and aripiprazole SC
	Fekete et al., 2021 [95]	Retrospective	Risperidone, venlafaxine	Total sample: 1555; patients < 18: 100	Patients < 18 treated with risperidone: 14.0; 4.0 (mean; IQR), 7–17 (range); patients < 18 treated with venlafaxine: 16.0; 2.0 (mean; IQR), 12–17 (range)	German database; ethnicity not stated	Not stated	Risperidone and venlafaxine dose-adjusted SC lower in adolescents than adults
	Piacentino et al., 2020 [96]	Retrospective	Aripiprazole, risperidone	140	14.2 ± 3.1 (mean ± SD), 5−18 (range)	Ethnicity not stated	Oppositional Defiant/Conduct Disorders	Higher aripiprazole and risperidone dose-adjusted SC in girls; lower risperidone active moiety SC in lower-weight patients
	Taurines et al., 2022 [109]	Prospective	Risperidone	64	15.6 ± 1.7 (mean ± SD), 11–18 (range)	Inpatients and outpatients; Ethnicity not stated	SSD	Higher SC associated with a higher risk of EPS. Preliminary indications for a lower TRR in this population
	Tini et al., 2022 [91]	Prospective	Sertraline	78	14.2 ± 2.4 (mean ± SD), 7–18 (range)	Ethnicity not stated	Obsessive–compulsive and Major Depressive Disorders	Strong linear positive dose–serum concentration relationship; significant effects of weight and co-medication; no association between dose or SC and side effects; higher doses and SCs are more effective in the treatment of the OCD
Elderly	An et al., 2021 [105]	Retrospective	Olanzapine	185	18–87 (range), 67.6% of the sample aged ≥56	Chinese population	Schizophrenia	Age-related ↓ N-desmethyl olanzapine SC
	Castberg et al., 2017 [99]	Retrospective	Clozapine, olanzapine, quetiapine, risperidone	11,968	18–100 (range)	Data from Norwegian database; ethnicity not stated	Not stated	Age-related ↑ dose-adjusted SC of clozapine, olanzapine, quetiapine, risperidone, particularly in women
	Deng et al., 2020 [104]	Retrospective	Olanzapine	884	38 ± 16 (mean ± SD), 13–90 (range)	Chinese population	SSD, BD	Age-related ↑ olanzapine SC and dose-adjusted SC
	Fekete et al., 2020 [95]	Retrospective	Risperidone, venlafaxine	Total sample: 1555, patients > 60 years: 428	Patients > 60 treated with risperidone: 73.0; 11.0 (mean; IQR), 61–92 (range); Patients > 60 treated with venlafaxine: 71.0; 13.0 (mean; IQR), 60–93 (range)	German database, ethnicity not stated	Not stated	Age-related ↑ risperidone dose adjusted SC
	Hansen et al., 2017 [48]	Retrospective	Venlafaxine	1177	45 (median), 34–59 (IQR range)	Danish database, ethnicity not stated	Not stated	↑ dose-adjusted venlafaxine SC in patients over 64
	Hefner et al., 2019 [108]	Retrospective	Amitriptyline, venlafaxine	34	69.7 ± 3.5 (mean ± SD), 65–78 (range) (data referred to the whole database from which data on the 34 patients included in the study are taken from)	Ethnicity not stated	Not stated	Correlation between amitriptyline and venlafaxine SC and QT prolongation in the elderly
	Huang et al., 2021 [78]	Retrospective	Amisulpride	121	35.83 ± 13.50 (mean ± SD)	Inpatients Chinese population	Schizophrenia	Age-related ↑ dose-adjusted SC of amisulpride; proposal of 600 mg/day as the maximum dosage of amisulpride in patients over 60
	Jönsson et al., 2019 [100]	Retrospective	Amisulpride, aripiprazole, clozapine, flupenthixol, haloperidol, olanzapine, perphenazine, quetiapine, risperidone, sertindole, zuclopenthixol, ziprasidone	Different n° of patients for each drug, ranging from 11,272 (olanzapine) to 225 (sertindole)	Different ages for each drug ranging from 31 (median, sertindole) to 50 (median, haloperidol)	Data from Norwegian database; ethnicity not stated	Not stated	Age-related ↑ dose-adjusted SC of amisulpride, aripiprazole, clozapine, haloperidol, olanzapine, perphenazine, quetiapine, risperidone, sertindole, zuclopenthixol, particularly in women
	Reeves et al., 2018 [101]	Prospective	Amisulpride	8	76 ± 6 (mean ± SD)	Outpatients; Caucasian, African, Asian	Very late-onset schizophrenia-like psychosis	D2 receptor occupancy over 40% and good clinical efficacy with an amisulpride dose of 50 mg/day
	Smith et al., 2018 [107]	Retrospective	Lamotrigine	534	40 (median), 18–95 (range)	Norwegian database, ethnicity not stated	Not stated	Age-related ↑ lamotrigine dose-adjusted SC, particularly in women carrying the UGT1A4*3 allele
	Tveit et al., 2020 [45]	retrospective	Citalopram, escitalopram, mirtazapine, sertraline, venlafaxine	806 (2007 cohort); 1932 (2017 cohort)	2007 cohort, age < 65: 41, 19 (median, IQR); age ≥ 65: 76, 13 (median, IQR); 2017 cohort, age < 65: 41, 22 (median, IQR); age ≥ 65: 79, 15 (median, IQR)	Norwegian database, ethnicity not stated	Not stated	Age-related ↑ citalopram, escitalopram, mirtazapine, sertraline, venlafaxine SC, especially in women. Relevant percentage of the sample exposed to concentrations above TRR
	Tveito et al. 2021 [106]	Retrospective	Paliperidone LAI	1223	PP1M: 41.1 (mean) PP3M: 44.3 years (mean)	Norwegian database, one-third of the sample aged > 50; ethnicity not stated	Not stated	Age-related ↑ paliperidone dose-adjusted SC for paliperidone LAI users, especially in women
	Tveito et al., 2018 [103]	Retrospective	Olanzapine oral and LAI	8288	45, 18–102 (median, range)	Data from Norwegian database; ethnicity not stated	Not stated	Age-related ↑ olanzapine dose adjusted SC for oral formulation but not LAI users
	Tveito et al., 2021 [102]	Retrospective	Zuclopenthixol oral and LAI	2044	Oral subgroup: 52.3 ± 17.5 (mean ± SD); LAI subgroup: 47.3 ± 15.4 (mean ± SD)	Data from Norwegian database; ethnicity not stated	Not stated	Age-related ↑ dose-adjusted SC of zuclopenthixol for both oral and LAI formulation; LAI users over 65 and with low CYP2D6 function at risk of high SC exposure

BD: bipolar disorder; EPS: extrapyramidal side effects; LAI: long-acting injectable; PP1M: 1-month paliperidone palmitate injection; PP3M: 3-month paliperidone palmitate injection; SC: serum concentration; SSD: schizophrenia spectrum disorders; TRR: therapeutic reference range.

**Table 7:** Original articles on the use of TDM in patients with abnormal body weight.

Study	Study Design	Drugs	n	Age	Sample Characteristics	Diagnosis	Outcome
An et al., 2021 [105]	Retrospective	Olanzapine	185	18–87 (range)	Chinese population	Schizophrenia	High body weight associated with ↓ olanzapine and n-desmethylolanzapine SC
Diaz et al., 2017 [53]	Retrospective analysis of data from RCT	Clozapine	47	45 ± 10 (mean ± SD), 28–62 (range)	Patients included in RCT; ethnicity: African Americans, Caucasians	Schizophrenia	Association between weight gain and ↑ SC
Kuzin et al., 2021 [110]	Retrospective	Clozapine	455	19–88 (range)	German database, in- and outpatients, ethnicity not stated	Not stated	High BMI associated with ↑ clozapine SC and C/D ratio
Methaneethorn et al., 2017 [111]	Retrospective	Valproic acid	206	39.3 ± 13.1 (mean ± SD)	Asian ethnicity	BD, manic episode	Reduced clearance in overweight patients
Schoretsanitis et al., 2018 [112]	Retrospective	Venlafaxine	737	45.4 ± 14.6 (mean ± SD)	German database, in- and outpatients, ethnicity not stated	SSD, mood disorders, other	Negative correlation between BMI and venlafaxine active moiety SC, O-desmethylvenlafaxine SC, and venlafaxine C/D ratio
Warrings et al., 2021 [113]	Retrospective	Amitriptyline, clozapine, doxepin, escitalopram, mirtazapine, quetiapine, risperidone, venlafaxine	1657 (whole sample)	18–92 (range)	In- and outpatients, ethnicity not stated	Not stated	Negative correlation between BMI and venlafaxine active moiety SC, O-desmethylvenlafaxine SC

BD: bipolar disorder; BMI: body mass index; C/D: concentration/dose; SC: serum concentration; SSD: schizophrenia spectrum disorders.

**Table 8:** Original articles on the use of TDM in patients with systemic inflammation or other medical conditions.

Study	Study Design	Drugs	n	Age	Sample Characteristics	Diagnosis	Outcome
Courlet et al., 2019 [119]	Retrospective	Escitalopram	110	48, 36–56 (median, IQR)	Patients living with HIV, control group of patients not affected by HIV	Not stated	Escitalopram SC below TRR in 56% of HIV + patients; no significant DDI with antiretroviral drug identified
Scherf-Clavel et al., 2020 [117]	Retrospective	Aripiprazole, olanzapine, quetiapine, risperidone	Aripiprazole: 30, olanzapine: 24, quetiapine: 166, risperidone: 45	18–85 (range)	In- and outpatients with CRP concentration ≥ 0.5 mg/dL	Not stated	Positive correlation between elevated CRP and quetiapine C/D
Zhang et al., 2021 [116]	Retrospective	Aripiprazole, clozapine, quetiapine, risperidone	Aripiprazole: 13, clozapine: 42, quetiapine: 21, risperidone: 36	16–76 (range)	Inpatients with respiratory tract infections	Not stated	Higher C/D ratios during infection for all drugs considered

C/D: concentration/dose; CRP: C-reactive protein; DDI: drug–drug interaction; SC: serum concentration; TRR: therapeutic reference range.

## Data Availability

Not applicable.
